# The Origins of NAFLD: The Potential Implication of Intrauterine Life and Early Postnatal Period

**DOI:** 10.3390/cells11030562

**Published:** 2022-02-05

**Authors:** Francesco Valentini, Giulia Rocchi, Umberto Vespasiani-Gentilucci, Michele Pier Luca Guarino, Annamaria Altomare, Simone Carotti

**Affiliations:** 1 Pediatric Unit, Sant’Andrea Hospital, Faculty of Medicine and Psychology, “Sapienza” University of Rome, Piazzale Aldo Moro 5, 00185 Rome, Italy; franzvalentini@gmail.com; 2 Unit of Food Science and Human Nutrition, Campus Biomedico University of Rome, Via Alvaro del Portillo 21, 00128 Rome, Italy; giulia.rocchi@unicampus.it; 3 Unit of Internal Medicine and Hepatology, Fondazione Policlinico Campus Biomedico of Rome, Via Alvaro del Portillo 21, 00128 Rome, Italy; u.vespasiani@policlinicocampus.it; 4 Gastroenterology Unit, Fondazione Policlinico Campus Biomedico of Rome, Via Alvaro del Portillo 21, 00128 Rome, Italy; m.guarino@policlinicocampus.it; 5 Unit of Microscopic and Ultrastructural Anatomy, Campus Biomedico University of Rome, Via Alvaro del Portillo 21, 00128 Rome, Italy; s.carotti@unicampus.it

**Keywords:** non-alcoholic fatty liver disease, gut microbiota, fetal programming, gut-liver axis

## Abstract

Fetal life and the first few months after birth represent a plastic age, defined as a “window of opportunity”, as the organism is particularly susceptible to environmental pressures and has to adapt to environmental conditions. Several perturbations in pregnancy, such as excessive weight gain, obesity, gestational diabetes mellitus and an inadequate or high-fat diet, have been associated with long-term metabolic consequences in offspring, even without affecting birth weight. Moreover, great interest has also been focused on the relationship between the gut microbiome of early infants and health status in later life. Consistently, in various epidemiological studies, a condition of dysbiosis has been associated with an increased inflammatory response and metabolic alterations in the host, with important consequences on the intestinal and systemic health of the unborn child. This review aims to summarize the current knowledge on the origins of NAFLD, with particular attention to the potential implications of intrauterine life and the early postnatal period. Due to the well-known association between gut microbiota and the risk of NAFLD, a specific focus will be devoted to factors affecting early microbiota formation/composition.

## 1. Introduction

Over the last twenty years, an origin during the developmental age has been increasingly proposed for many chronic non-communicable diseases. This period represents a plastic age, defined as a “*window of opportunity*”, since the organism is particularly susceptible to environmental pressures and has to adapt to environmental conditions [1]. This plasticity is greatest during fetal life and in the first few months after birth, which represents the phase of maximum acceleration of development. Barker’s theory, known today as “*The Developmental Origin of Health and Disease*”, suggests that adaptive responses in children may determine long-term disease risk in adults. Little attention has been paid to this causal relationship so far; actually, it could have profound implications for both research and public health.

Several studies have shown that prenatal, perinatal and postnatal events can have a strong impact on an individual’s metabolic health in the medium and long term. In particular, various perturbations in pregnancy, such as excessive weight gain, obesity, gestational diabetes mellitus (GDM) and an inadequate or high-fat diet, have been associated with long-term metabolic consequences in offspring, even without affecting birth weight. Extensive research has focused on the relationship between maternal nutritional status and subsequent metabolic diseases in offspring, such as obesity, type 2 diabetes (T2D) and non-alcoholic fatty liver disease (NAFLD).

In this context, recent estimates have indicated a 25% increase in the global prevalence of NAFLD, especially in the Middle East and South America and the lowest in Africa, which is therefore emerging as the most common liver disorder and the second most common cause of liver transplantation [2,3].

This article aims to summarize the current knowledge on the origins of NAFLD, with particular attention to the potential implications of intrauterine life and the early postnatal period. Due to the well-known association between gut microbiota and the risk of NAFLD, a specific focus will be devoted to factors affecting early microbiota formation/composition.

## 2. The Origins of NAFLD and Fetal Lipid Metabolism

In the mid-1980s Barker et al. first exposed the theory of the “*developmental origins of health and disease*”. Since then, even with the advent of epigenetics, growing importance has been given to the factors that during fetal life and early childhood can influence the development of pathologies from childhood to adulthood [4]. Indeed, fetal development represents a critical period in which exposure to environmental insults has permanent effects on the structure and function of the offsprings’ organs, tissues and body systems [5].

The “footprints” of the disease in pre- and peri-natal life have also been identified for NAFLD [6]. Along with metabolic syndrome and obesity, NAFLD is one of the most common metabolic diseases in pediatrics, affecting ~3–10% of children [7,8]. A retrospective study on 182 children with a histological diagnosis of NAFLD demonstrated that fetal programming-related factors, such as gestational age, birth weight, genetic polymorphisms and parental obesity, are correlated with the degree of fibrosis and steatosis [9]. These clinical associations have been further investigated with the aim of disclosing the involved pathways. Alterations in liver lipid metabolism both in intrauterine life and in the early postnatal period have been observed, revealing the footprints of NAFLD development already from the prenatal age [10].

**Figure 1:**
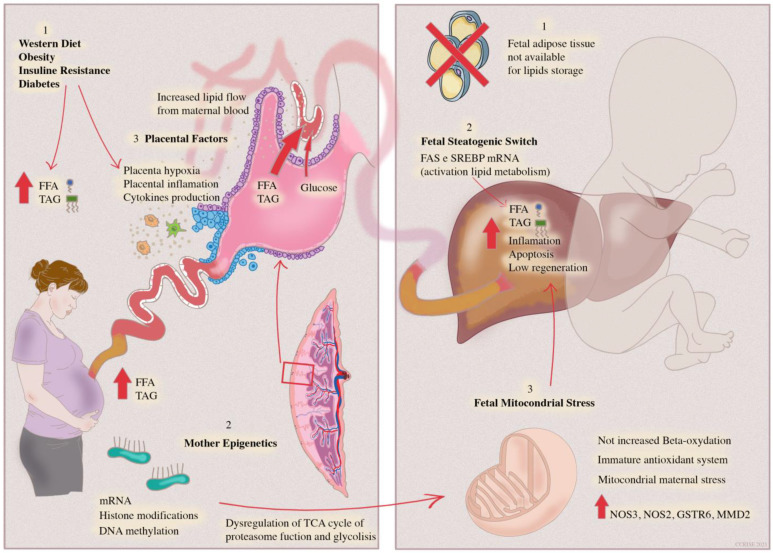
Mother–infant dyad. The steatogenic switch leading to adult NAFLD is triggered by pre-natal period factors. Mother’s dysregulated diet and comorbidities determine an increased flow of transplacental lipids leading to fetal lipid overload; lipids are stored in hepatocytes since fetal adipose tissue is unable to store lipids. Placental inflammation and hypoxia generated by maternal insulin resistance and obesity are transferred directly to the fetal liver through cord blood, leading to hepatic inflammation.

Fetal liver development begins at the fourth week of gestation and is closely related to maternal blood circulation and the placenta. The fetal liver typically bases its oxidative metabolism on carbohydrates, such as glucose, lactate and amino acids, while lipogenesis and lipid oxidation are suppressed, as suggested by the reduced expression of the related genes [11]. After birth, however, with the onset of breastfeeding, a fat-based metabolism emerges. The origin of NAFLD in the fetal period seems related to several factors: increased expression of liver enzymes involved in lipid metabolism, increased lipid load by trans-placental flow and increased oxidative stress due to intra-hepatic lipid accumulation (Figure 1) [12].

Hepatic steatosis and inflammation induced by mother’s factors are aggravated by an immature antioxidant fetal system and a low-beta oxidation rate, leading to mitochondrial stress and consequent hepatocyte apoptosis.

During gestation, the fetal liver directly receives about 50% of maternal blood flow through the branch of the umbilical vein that drains to the portal vein [13]. Therefore, the fetal liver, and in particular the right lobe, is anatomically positioned to be directly influenced by umbilical circulation and by the lipid load present in maternal blood. Experimental studies in animal models have explored whether the Western diet (high fat diet-HFD) or comorbidities like DM2 and obesity modulate fetal hepatic metabolism. Studies in non-human primates (NHP) have shown an increased hepatic expression of fatty acid synthase (FAS) and sterol regulating binding protein 1 (SREBP1) in the offspring of a diabetic and obese mother, suggesting an activation of fetal lipid metabolism [14]. The expression of genes related to mitochondrial and peroxisomal oxidation and to fatty acid transport was increased in the liver of 15-week-old offspring of mice fed a high-fat diet [15]. In this same study, rate-limiting enzymes of de novo lipogenesis (FAS) and TG synthesis (diacylglycerol acyltransferase—Dgat) were upregulated in the offspring’s liver, thus contributing to the severe hepatic steatosis observed at the histological level [15]. Notably, another study showed that fetal hepatic metabolism of non-human primates fed HFD undergoes a “steatogenic switch”; fetal hepatic deacetylase Sirtuin1 transcription, translation and activity were decreased, leading to an increase in enzymes involved in lipid metabolism and gluconeogenesis (PPARA, PPARG, SREBF1, FASN, SCD and CYP7A1) [16]. These data are in line with those of McCurdy et al., who found increased hepatic expression of gluconeogenic enzymes and transcription factors in the fetal liver of the offspring of both lean and obese mothers chronically consuming a HFD [17]. Therefore, lipid accumulation seems to be at least partially dependent on the activation of de novo lipogenesis, which is normally suppressed in the fetal liver. On the other hand, the liver is the only lipid storage organ during fetal life because, until the third trimester of pregnancy, the fetal adipose tissue is not able to receive back excess lipids from maternal placental circulation. Moreover, lipid oxidation pathways are not yet fully functional [11,18]. Indeed, triglyceride levels in the fetal liver of offspring of HFD primates were increased compared to the offspring of healthy controls [17]. Furthermore, in offspring of HFD primates, liver damage and steatosis are not reversible even after the introduction of a healthy diet in infants, and despite the fact that white adipose tissue has formed, suggesting that steatogenic priming has occurred. Conversely, in this same study, a change in maternal diet, from a high-fat to a normocaloric regime during pregnancy, results in liver levels of TG and expression of genes related to lipid metabolism in 1-year-old offspring that are quite comparable to those found in the liver of offspring of mothers fed a normocaloric diet [17].

Lipid oxidation plays a pivotal role in the progression from simple steatosis to steatohepatitis (Nonalcoholic steatohepatitis—NASH). However, the increase in lipid oxidation is not as much as the increase in lipid load and represents an ineffective adaptive response leading to oxidative stress, inflammation and hepatocyte apoptosis (Figure 1) [19].

Specifically, in an NHPs model, McCurdy et al. demonstrated that exposure to an HFD causes oxidative stress and promotes lipotoxicity and fetal liver damage. Lipid accumulation in the fetal liver has been associated with increased activation of stress-related genes (p-JNK), inflammation and circulating cytokines inducing NASH [17]. NAFLD-like changes found in fetuses from HFD mothers are resistant to dietary changes after weaning; in utero exposures to HFD and insulin resistance result in a steatogenic and inflammatory switch in the liver of 1-year-old NHPs [14].

Grant et al. observed a significant increase in the number of apoptotic cells in the fetal liver of offspring of HFD mice compared to controls; furthermore, when the same mice underwent subsequent pregnancy on a normocaloric diet, the degree of apoptosis in the fetal liver of the offspring was normalized [20]. In the fetal liver, the pro-inflammatory state typical of dysmetabolic conditions is not counteracted by an immature antioxidant system, inducing stress and mitochondrial damage [21].

A special focus on mitochondrial activity is present in many studies concerning the developmental origin of NAFLD (Figure 1). Indeed, mitochondria are the site of fatty acid oxidation, one of the most important liver functions affected by oxidative stress since the beginning of NAFLD. On the other hand, it is likely that mitochondria are mostly implicated in the inheritance of diet-induced maternal stress [22,23].

In a murine model, Bruce et al. showed that maternal fat intake contributes to the development of NAFLD in adult offspring because of inherited mitochondrial dysregulation. Mitochondrial damage induced a “proinflammatory priming effect” by increasing the expression of proinflammatory genes, such as Nos3, Nos2, Gstm6 and Lcn2, and related Crp genes, Mmd2, Tnfsf1 and Il-12b. Furthermore, mitochondrial dysfunction also caused lipid overload due to ineffective mitochondrial beta-oxidation, determining a “lipogenic priming effect” with changes extending to fetal and postnatal life [15]. The compromised activity of mitochondrial electron transport chain enzymes in the offspring liver was able to increase reactive oxygen species availability, promoting lipid peroxidation and inflammation, in turn making NAFLD more likely to develop in adulthood [15].

In the same study, Bruce and colleagues showed an upregulation of genes involved in fatty acids and triacylglycerol synthesis, despite unchanged gene expression of the rate-limiting enzyme involved in mitochondrial beta-oxidation, in the offspring of mothers fed a HFD compared to control progeny. These results demonstrate that mitochondria are unable to adequately provide the expected increase of beta-oxidation required by HFD metabolic demands. Consequently, intracellular acyl-CoA was directed toward TG synthesis pathways rather than beta-oxidation [15]. Furthermore, in the offspring of HFD mothers, even the number of mitochondria appears to be reduced, as demonstrated by the analysis of the number of mtDNA copies HFD [24].

Another main determinant of fetal steatosis is the damage induced and mediated by the placenta. The placenta regulates the availability of energy substrates to the fetus from maternal circulation [25]. Trophoblast cells modify the absorption and metabolism of glucose to maintain a constant concentration in the fetus. Conversely, lipids are transported differently according to their structure. FFAs are transported directly by the trophoblast from maternal to fetal blood, while triglycerides are transported only after hydrolysis, temporary storage and subsequent reesterification by trophoblast lipase [26,27,28].

In this way, despite women frequently exhibiting a considerably altered lipid profile during the third trimester of pregnancy, fetal blood lipid levels are maintained in a fairly normal composition range [29]. However, increased FFA levels have been found in umbilical cord blood samples of fetuses from mothers with gestational diabetes mellitus (GDM). Radeallii et al. demonstrated that trophoblast cells of GDM mothers show an expression of genes involved in fetoplacental lipid metabolism much more increased compared to glucose metabolism [25]. These data demonstrate that maternal insulin resistance has a greater effect on increasing transplacental lipid flow instead of glucose flow.

Furthermore, the placenta can induce steatogenic damage through inflammatory cytokines, oxidative stress and hypoxia (Figure 1). Several studies have shown that maternal HFD, obesity and GDM damage placental hemodynamics, leading to hypoxia [30,31]. Moreover, in the placental transcriptome of 16 women with GDM, an increased expression of proinflammatory and stress-related genes was found [32].

### 2.1. Evidence in Humans

Maternal overnutrition and obesity are dramatically associated with increased long-term risk for offspring obesity and pediatric NAFLD [33,34,35,36,37]; actually, in humans, the causal drivers of this association are still unclear. On the other hand, data regarding the effect of maternal NAFLD on infant health and pediatric hepatic metabolism are just starting to emerge. Data on human fetal liver metabolism are scarce due to the paucity of available samples. However, several studies have observed that hepatic lipid accumulation also occurs in human fetuses from mothers with DM or obesity. Patel et al. confirmed histopathologically that fetuses from diabetic mothers had a higher prevalence of steatosis and that the degree of steatosis was related to gestational age [38].

Modi et al. evaluated neonatal steatosis by analyzing hepatic fat with MR spectroscopy and found a direct correlation between maternal BMI at conception and the degree of neonatal steatosis [39]. MR spectroscopy was also used by Brumbaugh et al., who demonstrated that hepatic fat content in infants of diabetic and obese mothers is 68% higher compared to that in infants of healthy mothers [40]. Surprisingly, in these studies, the abundance of adipose tissue was not correlated with neonatal hepatic fat. These data suggest that, during fetal life, the accumulation of lipids in these two compartments follows different metabolic pathways and that the liver represents the most important lipid storage organ.

### 2.2. Epigenetics

Epigenetic changes in the liver during fetal life could determine a steatogenic imprint [12]. Different epigenetic mechanisms, including histone modifications, DNA methylation and miRNAs are involved in NAFLD development [41]. Puppala et al. showed that, in the fetal liver of baboons with obese mothers, the miRNAs involved in the transcription of lipid metabolism-related genes were significantly different compared to those in baboons with healthy mothers. The alteration of miRNA levels was correlated with dysregulation of the TCA cycle, proteasome function, oxidative phosphorylation, glycolysis and Wnt/β-catenin signaling pathways [42]. Similar results were obtained in a mouse model study, with reduced hepatic miR-122 and increased miR-370 levels in offspring of obese mothers compared to that of healthy controls. These findings correlated with decreased lipid oxidation markers (Cpt1a and Acadvl), and increased markers of triacylglycerol synthesis (Agpat and Gpam), respectively [43]. Furthermore, epigenetic changes have been correlated with both steatosis and inflammation/fibrosis. In a murine model exposed to an obesogenic diet during the gestational period and in the first weeks of life, the methylation pattern produced an increase in the transcription of genes linked to hepatic fibrosis and to activation of hepatic stellate cells, thus facilitating the progression from simple steatosis to NASH. In the same study, adult mice that had been exposed to an obesogenic diet during the gestational/perinatal period showed higher steatosis, inflammation and fibrosis when exposed to an obesogenic diet during adult life compared with adult mice treated with an obesogenic diet in adult life but having received normal feeding during the gestational/perinatal period. Thus, methyloma modification in neonatal age can also affect adult life [44].

Dudley et al. pointed out that an obesogenic diet during pregnancy induces hypomethylation of the cyclin-dependent kinase inhibitor 1a (CDKN1a), an inhibitor of the hepatocyte cell cycle. This epigenetic modification and consequent upregulation of CDKN1a, well described in adult liver diseases, represents an early hepatic dysfunction of the newborn that could predispose it to NAFLD in adulthood, impairing the capacity of the liver to deal with stressing conditions [45].

### 2.3. Autophagy

On the other hand, several studies have shown that intrauterine growth restriction (IURG) leads to an increased risk of developing NAFLD in adult life [46,47]. A recent experimental study in mice evaluated the effect of intrauterine and early postnatal calorie restriction (IPCR) on the function of the hepatic autophagic machinery, both in the neonatal period and in adult life. In the perinatal period, mice exposed to IPCR show impaired autophagy, which determines the accumulation of ubiquitinated proteins and lipid oxidative products. Conversely, in adult life, when exposed to a normal diet, these animals display an increase in hepatic autophagy compared to healthy controls [48]. These findings are supported by epidemiological studies in humans, such as those on children conceived during the Dutch “*winter of hunger*” (1944–1945) or during the great Chinese famine (1950–1960), where maternal malnutrition and decreased birth weight were directly correlated with obesity, diabetes, hyperlipidemia, NAFLD and metabolic syndrome in adulthood [49,50].

## 3. The Infant Gut Microbiota

Great interest has also been focused on the relationship between the gut microbiome of early infants and health conditions in adult life.

The human gastrointestinal tract is colonized by a complex microbial community, defined as the gut microbiota, made up of trillions of microbial cells, whose taxonomic abundances and metabolic activities—known for establishing complex trophic relationships with the human host organism—are considered important to ensure intestinal homeostasis and health [51]. The functions of the gut microbiota provide several benefits to the human host, including digestion of food, synthesis of short-chain fatty acids (SCFA), vitamins and other essential substances. Other key functions of the gut microbiota include ensuring that the body has adequate defense against harmful microorganisms, regulating the immune response and promoting endocrine homeostasis [1]. Consistently, in various epidemiological studies, a condition of dysbiosis, or reduced richness and diversity of intestinal bacterial taxa, has been associated with an increase in the inflammatory response and metabolic alterations of the host, with important consequences on the intestinal and systemic health of the unborn child [1,51,52]. Therefore, the available experimental data support the long-term health benefits induced by the infant intestinal microbiota, mainly through the modulation of risk factors related to particular health conditions in adults [53].

The biogenesis of the intestinal microbiota, which occurs at birth and in the first years of life in a healthy human intestine, is a dynamic and non-random process driven by both evolutionary and environmental signals capable of modulating the composition and temporal stability of the interactions, positive and negative, among the main microbial taxa and between them and the human host in childhood [54,55].

It has been reported that this process of developing and maturing the infant gut microbiota to reach its “*adult-type*” form is influenced by various perinatal conditions, such as the mode of delivery, the type of breastfeeding, and the use of antibiotics. Moreover, the mother’s diet, age, metabolic status, family genetics and lifestyle have also been reported to have a strong impact on the infant microbiota [54,56]. Until now, it was believed that the first populations of microbial cells of the gastrointestinal tract were acquired only at the time of birth through the modality of delivery, vaginal and cesarean, thus reaching their maximum density during the infancy of the host [55]. However, the increasing number of observations describing the presence of bacteria and/or bacterial DNA in the placenta, amniotic fluid, umbilical cord and meconium have allowed us to dispel the common historical dogma of sterility at birth and question the in utero colonization theory [57,58,59,60], assuming a vertical maternal–fetal transmission pathway of intestinal microbial communities. Furthermore, it would seem that the type and methods of acquisition of the first colonizing inoculum strongly depend on the maternal health status, on the body mass index (BMI) and body weight, on the gestational age, and above all on intrauterine and postnatal nutrition [54]. Understanding how environmental factors and nutritional exposure, including breast milk, affect the assembly and development of both maternal and infant microbial communities may help to identify targeted interventions during pregnancy and lactation in infants born from mothers with obesity to slow the transmission of metabolic risk to the next generation.

### 3.1. Nutritional Exposures and Infant Gut Microbiota Development

It is almost certain that the first environmental experiences, including the supply of nutrients during intrauterine and extrauterine life, and the development of gut microbiota might have long-term implications on the manifestation of chronic-degenerative disorders in adulthood. As previously mentioned, the gut microbial ecosystem plays an important role in human health. Early life provides a unique window of opportunity for modulating the gut microbiota to promote long-term health. Alterations and aberrations in its composition and function during neonatal age, as well as in infancy up to 2 years of age, have been associated with various pediatric disorders and diseases in old age [61,62]. Therefore, it can be assumed that the early gut microbiota contributes to the onset and progression of the disease and that the foundation for a stable adult-type gut microbiota must be laid as early as during the neonatal period and childhood. This concept explains the need for a thorough understanding of the composition and development of the infant microbiota, the interactions of the microbiota members with each other and with the infant host to maintain intestinal homeostasis and to prevent the onset of disorders and/or diseases at a future age. The main perinatal and postnatal factors capable of modulating the composition and activity of the infant intestinal microbiota and susceptibility to pediatric NAFLD are reported below.

*Breastfeeding*: Breastfeeding, in addition to supporting the nutritional aspects, growth and development of babies, has profound health benefits for babies, such as a reduction in the rates of childhood obesity, allergies, infection and diabetes mellitus [63]. The type and duration of breastfeeding in early infant life may have deep effects on the risk of NAFLD, even if there is not much data on the relationship between early feeding and subsequent NAFLD. In an observational study, Nobili et al. investigated the association between breastfeeding and NAFLD phenotype in children with NAFLD and found that earlier feeding habits might affect the clinical expression of NAFLD, with a long-term protective effect of breastfeeding on NASH (OR, 0.7; 95% CI, 0.001–0.87) and liver fibrosis (OR, 0.86; 95% CI, 0.75–0.98) from 3 to 18 years later, which was independent of the neonatal characteristics of the children [64]. Furthermore, Ayonrinde et al. reported an inverse association between the duration of breastfeeding and the age of introduction of formula in the subsequent diagnosis of NAFLD [65]. Specifically, the authors observed that infants who were breastfed for less than 6 months before starting formula milk were at least 70% more likely to develop NAFLD. While exclusive breastfeeding for at least 6 months without supplementation with supplemental milk until after 6 months of age, reduced the odds of a diagnosis of NAFLD by nearly 40% after adjustment for pre-pregnancy maternal obesity and obesity during adolescence, regardless of age at introduction of complementary solid food and Western dietary pattern at 17 years of age. These data suggest that infants who breastfed for <6 months before introducing formula or babies who had obese mothers in early pregnancy were much more likely to develop NAFLD later in life. Indeed, compared to artificial feeding, some trophic nutritional factors present in human milk can interact with hepatocytes within a neurohormonal environment [66]. This is mediated by many regulatory systems, such as receptors activated by peroxisome proliferation (PPAR-α and -γ) and docosahexaenoic acid (DHA), which could act as a PPAR agonist, both directly and indirectly, involved in reducing experimental hepatic fibrogenesis [67,68]. In addition, the type of breastfeeding is very important for the maturation and development of the intestinal microbiota of the unborn child. Breastfeeding and formula milk are known to determine early microbial colonization and, consequently, to greatly influence the composition and activity of the neonatal intestinal microbiota and the gastrointestinal function. The differences in the composition and abundance of intestinal bacterial taxa between breastfed and formula-fed infants have been well documented in multiple epidemiological studies. Breastfeeding has been shown to increase specific taxa, such as *Bifidobacterium* spp., while decreasing *Clostridium* spp., *Bacteroides* spp., and *E. coli*; opposite findings are reported with formula feeding [69,70,71,72,73]. Breastfed infants tend to have less gut diversity and microbial richness than formula-fed infants, but they also have more consistent associations with individual taxa that have previously been linked to early life diet and health outcomes. In particular, in breastfed infants, a greater abundance of *Bifidobacterium* and *Lactobacillus,* which bring pleiotropic benefits to the infant human host, mainly through the production of SCFAs from microbial fermentation processes, has been reported [74]. From a microbiological point of view, although the knowledge of commensal bacteria present in the milk of healthy women is very limited, staphylococci, streptococci, micrococci, lactobacilli and enterococci have been isolated from this substrate [75,76,77,78]. Additionally, several studies have shown that breast milk provides a mix of nutrients [e.g., lactose, glycans, casein, α-lactalbumin, lactotransferrin, serum albumin, orosomucoid, amino acids, lysozyme, lipase, growth factors, triglycerides, arachidonic acid (AA), docosahexaenoic acid (DHA), vitamins, minerals, trace elements, etc.] and pro-microbial and antimicrobial agents (e.g., immunoglobulins, immunocompetent cells or different antimicrobial compounds), which play a pivotal role in selectively modeling the growth and function of beneficial microbes, such as to favor the maturation of the gastrointestinal tract of the unborn child and to promote a regulatory and more “tolerogenic” immune system [79]. In particular, human milk glycans, such as human milk oligosaccharides (HMOs), are a major source of important immune and growth factors necessary for initial microbial colonization. It is generally accepted that the metabolism of HMOs by the gut microbiota has a significant prebiotic effect [80], capable of enriching *Bifidobacteria* populations [81], and that the type and amount of oligosaccharides that infants receive from their mothers depend on the genotype and phenotype of the mother [82,83].

*Weaning and Diet:* Weaning and infant eating habits can also have a major impact on the metabolic health of the liver. Animal models suggest that a high-fat diet in early childhood both during and after weaning may exaggerate the risk and severity of pediatric NAFLD, regardless of maternal obesity during gestation and lactation [15,17,84]. Furthermore, there is a lot of evidence in the literature that supports how the maternal diet during pregnancy and lactation contributes to shaping the intestinal microbiota of the offspring, with short- and long-term effects on health. The maternal gut microbiota differs among women with normal weight and obesity, particularly in the second half of pregnancy, with overweight women showing increases in the Firmicutes phylum (*Staphylococcus*), as well as increases in some Proteobacteria (*Escherichia coli*), and these differences are associated with increased neonatal birth weight [85,86]. One mechanism by which maternal microbes in obesity could affect the developing fetus is through elevated levels of microbe-derived plasma endotoxin; this may increase gut translocation of bacteria-derived products across the intestinal mucosa, which could contribute to systemic and placental inflammation and insulin resistance [87]. One of the most relevant investigations to examine the role of the maternal diet in the constitution of the juvenile microbiome was performed in 2014 [88]. In this study, carried out in a primate model (*Macaca fuscata*), two isocaloric diets during pregnancy and lactation, with a different energy intake of fats [high-fat diet (HFD), 36% fat and control diet (CTD), 13% fat], were compared in order to assess whether maternal high-fat diet structures a dysbiotic gut microbiome later in life in the offspring, even following a post-weaning control diet at 1 year of age. The researchers, regardless of the lean, obese or insulin resistant maternal phenotype, observed a clear separation between the early microbial profiles of the two groups, characterized by reduced species richness and diversity in the microbiota of offspring whose mother had been fed with the HFD vs. CTD. Furthermore, they observed that this microbial alteration was only partially corrected by a weaning control diet, suggesting that early changes in gut microbial communities may persist over time and are most likely due to maternal dietary exposure during gestation and breastfeeding. In summary, these data support the idea that diet most likely drives “dysbiosis” in primates and suggest that gut ecology may be structured across generations by multiple modalities, including maternal diet [88]. Subsequently, similar to the primate models, Chu et al. observed that independent from maternal BMI, maternal HFD is associated with distinct changes in the neonatal gut microbiome at birth that persist through 4–6 weeks of age. These findings underscore the importance of counseling pregnant mothers on macronutrient consumption during pregnancy and lactation [89]. Another study carried out on a cohort of 323 mother–infant pairs to determine the association between diet during pregnancy and the infant intestinal microbiome showed that a maternal diet high in vegetables and low in processed meats and deep-fried foods was positively associated with an increase in Shannon diversity index and richness, and a reduction of bacterial taxa, such as *Bacteroides* spp. and *Clostridium* spp. After adjustment for demographic variables, the maternal diet was also associated with an increase in *Lactobacillus* in infant stool samples collected between 3 and 6 months of age [74]. This persistent effect of the early-life diet suggests that maternal diet exposure during gestation and breastfeeding, in particular fat intake and caloric density, could have an impact on the formation and development of the childhood microbial community with long-lasting effects, and advice to investigate the etiology of many chronic disorders of adulthood in the pre- and peri-natal period. Indeed, taken together, these data suggest that, if aiming to reduce the risk of NAFLD during adolescence and adulthood, it is necessary to implement preventive strategies before birth, encouraging the achievement or maintenance of a normal maternal BMI before conception and promoting exclusive breastfeeding during lactation.

### 3.2. Gut-Liver Axis, Microbial Dysbiosis and Implications of Pediatric NAFLD

It is becoming increasingly clear that the gut microbiome in newborns and infants plays a significant role in gut health and child development. Alteration of the early infant gut microbiome has been correlated with the development of metabolic diseases, childhood obesity and autoimmune conditions, including asthma, allergies and more recently type 1 diabetes [90,91]. To date, the definition of dysbiotic and/or eubiotic intestine is still much debated in the scientific community. Based on the observations reported, the Shannon diversity index and richness (total number of taxa identified) were associated with positive health outcomes on gut microbiota composition, as well as the abundance of Lactobacillus spp and Bifidobacterium spp. In adult populations, NAFLD is associated with less bacterial diversity, increased abundance of the phylum Bacteroidetes, Ruminococcus, Lactobacillaceae, Proteobacteria and *E. coli*, and decreased abundance of the phylum Firmicutes and Actinobacteria, Oscillobacter, Prevotella, *Faecalibacterium prausnitzii* and Coprococcus compared with non-NAFLD controls. An increase in the Bacteroidetes:Firmicutes (B:F) ratio—due to increased abundance of the phylum Bacteroidetes and decreased abundance of Firmicutes—is sometimes reported as a dysbiotic marker that characterizes NAFLD [92]. Despite the absence of a consistent dysbiosis signature, all pediatric studies of NAFLD-associated gut microbiota typically describe decreased α-diversity (richness and evenness), significantly altered β-diversity, and significant differences in the abundance of bacteria at the phylum, class, family or genus level, compared with the microbiota of appropriate control subjects [93]. Regarding specific bacteria, the abundance of *Faecalibacterium prausnitzii* differs between obese children with and without NAFLD and a reduced abundance of Oscillospira has been confirmed in several studies of NAFLD in children [93].

It is known that antibiotics, cesarean section, and infant formula feed alter patterns of microbial acquisition and succession during the first 2 years of childhood [54]. The development of the microbiome from birth might have important long-term clinical implications in NAFLD. There is evidence to suggest that susceptibility to postnatal NAFLD is strongly associated with an early condition of intestinal dysbiosis [94] and impaired metabolic function of the infant intestinal microbial population, mainly the production and use of short-chain fatty acids (SCFA) and bile acids (BA) [95]. Bile acids mediate communication between the liver and intestines, and an altered bile acid composition and metabolism have been reported in the progression of NAFLD. In the intestine, bile acids undergo modifications by deconjugation and dehydroxylation by intestinal bacteria with the production of secondary bile acids. Secondary bile acids can influence, in turn, the growth and regulation of the intestinal microbiota and interact with various nuclear receptors, e.g., farnesoid X receptor (FXR) and G protein-coupled membrane receptor 5 (TGR5), which control various metabolic and immune pathways in the host. This microbial modification of bile acids is an important mechanism by which the microbiota can interact with the host and affect not only liver disease but also other organs and metabolic pathways. Therefore, early intestinal dysbiosis could contribute to the progression of NAFLD by modifying bile acids and their derivatives and by regulating the metabolic signaling pathways of FXR and TGR5 involved in NAFLD.

Absorbed SCFAs are delivered to the liver via the portal vein, where they contribute to energy metabolism by entering the tricarboxylic acid cycle to generate ATP and energy [96]. The involvement of SCFAs in the development of NAFLD could result from their association with fatty acid synthesis and gluconeogenesis. According to some evidence, greater production of acetate, a precursor for the synthesis of fatty acids, in the liver causes the accumulation of triglycerides, while propionate favors gluconeogenesis, contributing to weight gain [97,98]. Rau et al. suggested that elevated fecal levels of acetate and propionate, in association with an increased abundance of SCFA-producing bacteria, are associated with a decrease in regulatory T cells and a higher T helper 17 cell, immunological features involved in progression by NASH [99].

It is known that obesity is associated with dysbiotic gut microbiota, with decreased diversity and an increased *Firmicutes/Bacteroidetes* ratio [100]. According to Turnbaugh et al., the cecal content of SCFAs in obese mice showed in-creased levels of acetate and butyrate [101]. Similar results were also observed by Schwiertz et al., In which obese individuals had a higher total fecal concentration of SCFAs than lean individuals [102]. A prospective, observational, and cross-sectional study, carried out by Schwimmer et al. with the aim to analyze the microbiomes of children with and without NAFLD/ NASH, revealed that the fecal microbiomes of children with NAFLD had less α-diversity than controls (3.32 vs. 3.52), and that this reduced diversity was even more evident in those with NASH (2.97) [103]. Furthermore, the latter showed a greater enrichment of genes for the biosynthesis of lipopolysaccharide (LPS), genes involved in flagellar assembly, and observed that subjects with more severe fibrosis had a high abundance of *Prevotella copri.* These findings suggest *P. copri*’s adaptive ability to thrive in a proinflammatory gut environment to its advantage and exacerbate liver damage during NAFLD progression through the production of hydrogen peroxide and thioredoxin. Therefore, the authors hypothesized that a loss of diversity and an increase in *P. copri* abundance may be causal factors influencing NAFLD [103].

Several studies have also proposed mechanisms by which gut microbiota dysbiosis contributes to liver damage involved in the pathogenesis of NAFLD. The contributions of overgrowth of intestinal gram-negative bacteria, increased intestinal permeability, bacterial translocation, and elevated serum lipopolysaccharide (LPS) levels have been demonstrated in NAFLD [103,104,105,106,107]. In patients with NAFLD/NASH, gut dysbiosis promotes insulin resistance and hepatic de novo lipogenesis, and it also increases intes-tinal permeability, which promotes chronic PAMP exposure and oxidative stress [104]. Patients with NAFLD present increased circulating levels of LPS, and the higher acti-vation of hepatic TLR signaling is associated with inflammation and fibrogenesis [105]. Indeed, endotoxin/TLR4 signaling contributes to the development of fibrosis and pro-gression to cirrhosis through hepatic stellate cell activation [106]. Furthermore, as pre-viously cited, gut microbiome dysbiosis in NAFLD could affect the conversion of pri-mary bile acids into secondary bile acids. It has been observed that the bacteria able to make this transformation are decreased in the NAFLD cirrhosis fecal samples, with a higher level of Enterobacteriaceae and lower Ruminococcaceae, Lachnospiraceae and Blautia (with a 7_-dehydroxylating activity) abundances [107]. From an epigenetic and microbiome perspective, a study conducted to examine maternal overnutrition caused an exacerbation of postnatal hepatic responses in the mice C57BL6/J offspring with in-creased pro-inflammatory and pro-fibrogenic gene expression, persistent epigenetic changes in key genes involved in hepatic fibrosis and lipid accumulation (Fgf21, Ppargc1β, VWF, Ephb2), reduced α-diversity in gut microbiota profiles and an associa-tion between serum ALT levels and Coprococcus, Coriobacteriacae, Helicobacterioce-ae, and Allobaculum, indicating that maternal HFD negatively alters the epigenetic and gut microbiome pathways of the offspring that favor the development of NAFLD and its progressive consequences [108]. A summary table of some studies regarding dysbiosis and hepatic injury related to different nutritional exposure are shown in Table 1.

## 4. Conclusions and Future Directions

This review presented available evidence that points to the relevance of intrauterine and early postnatal life for the overall risk of NAFLD development and severity during the entire lifespan. It is important to know and to transmit a simple message, e.g., that the metabolic health status of the mother is somehow transmitted to the offspring not only in terms of genetic predisposition but also through epigenetic and circulating mediators that can be directly controlled and modified, among which the microbiota plays an important role. Consistently, eating behaviors and other determinants occurring during infancy and childhood can have a profound impact on metabolic health during adult life. The better we understand these “metabolic messages”, the higher our possibility to provide parents with more practice and specific recommendations aimed at preventing NAFLD and other dysmetabolic conditions.

## Figures and Tables

**Table 1:** Studies on gut microbiome dysbiosis and hepatic damage of offspring related to different nutritional exposure during intrauterine life and postnatal period. (↓: reduction; ↑ increase).

Condition		Treatment	Main Results	Ref.
**Animal**	Primate model *(Macaca fuscata)*	Diet during pregnancy: HFD (36% fat) vs. Control diet (13% fat)	[↓] Species richness and diversity These microbial alterations were only partially corrected by a weaning control diet at age 1	[88]
	C57BL6/J *mice*	Diet during pregnancy: HFD (45% fat) vs. Control diet (17% fat)	[↑] Body weight, relative liver weight and immune cell infiltration in liver biopsy [↑] Upregulated genes involved in fibrosis (*Cidea*, *Mmp12*) and inflammation (*Ly6d*); an altered methylation patterns of *Fgf21, Ppargc1β, VWF, Ephb2* genes [↓] α-/β-diversity, Coriobacteriaceae, Peptococcaceae, *Ruminococcus*, *Turibacter, Oscillospria*	[108]
	Swiss *mice*	Diet during pregnancy: HFD (45% fat) vs. Standard diet (9% fat)	In offspring from HFD mother group: [↑] Body weight, fat mass, hepatic triglycerides levels and large lipid vacuoles within hepatocytes [↑] Serum insulin, TNFα and interleukin 1β [↑] JNK, I kappa B kinase phosphorylation and PEPCK expression in the liver [↓] Basal p-ACC level and insulin signaling in the liver [↓] Hormone-sensitive lipase phosphorylation (*Ser565*) in epididymal adipose tissue	[109]
**Human**	Healthy pregnant woman	Diet during pregnancy: High vegetable diet	[↑] Shannon diversity index [↑] *Lactobacillus* spp. [↓] *Bacteroides* spp. and *Clostridium* spp.	[74]
	Healthy pregnant woman	Diet during pregnancy: HFD (43.1% fat) vs. Control diet (24.4% fat)	PCoA revealed that the microbiome of the neonatal stool at birth (meconium) clustered differently by virtue of maternal gestational diet. [↓] *Bacteroides* spp. in the neonates at birth exposed to a maternal gestational HFD that persisted to 6 weeks of age	[89]
	Children with and without NAFLD/NASH	-	[↓] α-diversity in NAFLD and NASH groups [↑] abundance of *P. copri* [↑] Genes of LPS biosynthesis and flagellar assembly were significantly enriched in microbiomes from children NASH	[103]

## Data Availability

Not applicable.
